# Isolated traumatic head injury in children: Analysis of 276 observations

**DOI:** 10.4103/0974-2700.76831

**Published:** 2011

**Authors:** Mabrouk Bahloul, Hedi Chelly, Anis Chaari, Imen Chabchoub, Sondes Haddar, Leila Herguefi, Hassen Dammak, Chokri Ben Hamida, Hichem Ksibi, Hatem Kallel, Noureddine Rekik, Mounir Bouaziz

**Affiliations:** Medical Intensive Care Unit, CHU H Bourguiba, Sfax, Tunisia; 1 Department of Radiology, CHU H Bourguiba, Sfax, Tunisia; 2 Department of Pediatrics, CHU H Chaker, Sfax, Tunisia

**Keywords:** Acute head injury, children, intensive care unit, motor-vehicle crash, prognosis, trauma

## Abstract

**Background:**

To determine predictive factors of mortality among children after isolated traumatic brain injury.

**Materials and Methods:**

In this retrospective study, we included all consecutive children with isolated traumatic brain injury admitted to the 22-bed intensive care unit (ICU) of Habib Bourguiba University Hospital (Sfax, Tunisia). Basic demographic, clinical, biochemical, and radiological data were recorded on admission and during ICU stay.

**Results:**

There were 276 patients with 196 boys (71%) and 80 girls, with a mean age of 6.7 ± 3.8 years. The main cause of trauma was road traffic accident (58.3%). Mean Glasgow Coma Scale score was 8 ± 2, Mean Injury Severity Score (ISS) was 23.3 ± 5.9, Mean Pediatric Trauma Score (PTS) was 4.8 ± 2.3, and Mean Pediatric Risk of Mortality (PRISM) was 10.8 ± 8. A total of 259 children required mechanical ventilation. Forty-eight children (17.4%) died. Multivariate analysis showed that factors associated with a poor prognosis were PRISM > 24 (OR: 10.98), neurovegetative disorder (OR: 7.1), meningeal hemorrhage (OR: 2.74), and lesion type VI according to Marshall tomographic grading (OR: 13.26).

**Conclusion:**

In Tunisia, head injury is a frequent cause of hospital admission and is most often due to road traffic injuries. Short-term prognosis is influenced by demographic, clinical, radiological, and biochemical factors. The need to put preventive measures in place is underscored.

## INTRODUCTION

Traumatic brain injury is the most common cause of death and acquired disability among children and young adults in developed countries and, even when adequate treatment is provided, traumatic head injury commonly causes neuronal loss.[1] The underlying pathophysiology highlights the importance not only of the primary injury, but also of the secondary processes occurring after injury, which may lead to cerebral hypoxia and ischemia.[2] Secondary brain injury is the leading cause of in-hospital deaths after traumatic brain injury.[2,3] Moreover, the outcome of childhood head injury varies from center to center depending on the availability of modern neurosurgical and neuroradiological facilities and qualified expertise.[4,5] In Tunisia, each year, nearly 13,000 victims of motor-vehicle crashes are recorded, and about 1500 patients die according to the National Guard Statistical Data.[1] Pediatric morbidity and mortality due to head trauma are increasing because of high rate of road traffic injuries. Survivors are susceptible to irreversible neurological damage that represents an important socioeconomic problem.[6,7] Head injury is the most frequent cause of mortality and morbidity in childhood. Finally, the prognosis may be influenced by the presence of extracranial pathology. However, the impact of isolated traumatic head injury on children outcome was rarely studied.

In the Sfax area (South Tunisia), all cases of severe traumatic head injuries are admitted in the medico-surgical intensive care unit (ICU) where specific monitoring tools (jugular venous saturation, intracranial pressure monitoring, and transcranial Doppler sonography) are not available.

The study was designed to evaluate the outcome of isolated traumatic head injury in children referred to our medico-surgical ICU, and to define simple predictive factors which can be used in routine practice in general ICUs as an indicator of poor prognosis.

## MATERIALS AND METHODS

This study was approved by an Internal Review Board. All consecutive patients with isolated traumatic brain injury, aged less than 15 years and admitted to ICU of Habib Bourguiba University Hospital during the 8-year period from 1997 to 2004, were included in this study. The data were recorded from the patient clinical notes with multiple contributors. Our department is a 22-bed medical surgical ICU in a teaching hospital of 510 beds that serves as first-line medical center for an urban population of 1 million inhabitants and as a referral center for a larger population coming from south Tunisia. The total number of admissions in our unit is about 1200 per year. In our department, three beds are reserved to pediatrics intensive care.

Patients were admitted directly from the scene of the accident within 6 h of injury. They were all examined and scored according to Glasgow Coma Scale (GCS) Score on arrival and underwent computed cerebral tomography (CT) scan as soon as feasible.

The patients’ medical files were retrospectively reviewed, and the following data were so extracted: age, gender, vital signs (heart rate, respiratory rate, systolic, and diastolic blood pressure), body temperature in °C (temp), Glasgow Coma Scale Score(GCS score),[8] Injury Severity Score (ISS),[8,9] Pediatric Trauma Score (PTS),[8,10] and Pediatric Risk of Mortality (PRISM) Score.[8,11] Others were causes of injury, pupil response, motor deficit, convulsion, use of mechanical ventilation, the presence of shock or arterial hypotension,[12] cardiac arrest, fluid intake volume, brain CT-scan result, and use of catecholamines (dopamine, dobutamine, epinephrine).

Before 2005 norepinephrine is not available in our ICU, so it was not reported in our study. Biochemical parameters measured on admission and during the ICU stay were arterial blood gases and acid–base state, hemoglobin concentration, platelet counts, serum glucose and sodium levels, blood urea and urine-specific gravity.

Plain radiographic studies of the neck were performed in all patients. Cranial CT-scan was done in all but four patients due to unavailability of CT-scan, for these four patients a brain magnetic resonance imaging (MRI) was performed on admission. The CT-scan findings were axed on the presence or absence of hematoma (whetherextradural, subdural, or intracerebral), meningeal hemorrhage, cerebral edema, cerebral contusion, pneumocephalus, intracranial mass lesion, and herniation. In addition, the cranial CT-scan results were stratified according to the “Traumatic Coma Data Bank Computed Tomography Classification” for Severe Head Injury”[13,14] The cerebral CT-scan classification was performed by an university radiologist.

Neurological state was assessed using the GCS score at the site of accident (by the medical doctor of pre-hospital care system) and again on hospital arrival before the use of sedative but after resuscitation: the preintubation GCS (used in our analysis). All patients were intubated, ventilated, and received sedation with thiopental sodium 50 mg /kg/day or with combined fentanyl–midazolam as necessary. Patients with diabetes mellitus and/or the use of glucose containing fluid given intravenously were registered. Corticosteroids were not used for the treatment of cerebral edema. In our ICU, the head bed kept elevated was used in all patients. Mannitol was used when cerebral CT-scan showed cerebral edema and/or herniation; however, hypertonic saline is not used in our practice. Mild hyperventilation (PaCO_2_ = 30–35 mmHg) was applied in all patients with severe traumatic head injury and requiring mechanical ventilation. Before January 2000, barbiturate therapy (thiopental sodium 50 mg/kg/day) was used in all patients admitted for severe traumatic head injury requiring mechanical ventilation. Nevertheless, this therapy was abandoned since this date. In our practice, anticonvulsants are used only if the patient developed seizure, hypothermia therapy as well as decompressive craniectomy was not used in our study. All patients with suspected intracranial pressure elevation and patients with herniation syndrome received mannitol. In our ICU, therapies were directed by repeated CT-scan.

For each patient, recorded for the study were mean of all daily Na, K and blood sugar level(BSL) and the peak/trough results. In addition, we have recorded the development of secondary systemic insults (SSI) on admission and during ICU stay. SSI were divided into subgroups of respiratory (hypoxemia, hypercapnia, and hypocapnia),[14,15] circulatory (hypotension or arterial hypertension),[16,17] metabolic/electrolytic SSI (anemia, hyper or hypoglycemia, hyponatremia, and diabetes insipidus),[14,18,19] and hyperthermia. During the ICU stay, all complications were recorded: nosocomial infections,[20] pneumonia,[20,21] tract urinary infection,[20,21] meningitis,[20] and septicemia.[20,22]

Glasgow Outcome Scale (GOS)[12,23] was performed after hospital discharge by ICU and pediatric physicians (in most cases). When extracranial pathology was suspected, appropriate investigations were performed. All clinical, biological, and radiological parameters and relevant therapeutic measures were registered on admission and during the ICU stay. All patients with confirmed extracranial pathology were excluded from this study.

### Statistical analysis

Categorical data were expressed in proportion and subgroups (survival and death) and were analyzed by the χ^2^ test. Continuous variables were expressed as means (±SD) and subgroups evaluated by Student’s *t*-test. In this analysis, to study the influence of age on outcome, we compared the mean age between survivors and nonsurvivors.

Risk factors were evaluated in univariate analysis and by multivariate analysis by a multiple logistic stepwise regression procedure. Odds ratios were estimated from the *P* coefficients obtained, with respective 95% confidence intervals (95% CI).

PRISM, PTS, ISS, and GCS Score were used to predict mortality and were analyzed using receiver operating characteristic (ROC) curves. The area under the ROC curve which was estimated by the method of Hanley and McNeill[23,24] provides a measure of overall mortality of the test. For comparable data, a *P* value less than 0.05 was considered as statistically significant.

## RESULTS

During the study period, 454 children were admitted in our ICU for traumatic head injury. Two hundred and seventy-six patients had an isolated traumatic head injury; they were all included in this study. Victims were rescued and brought to our hospital either by fire fighters or by a pre-hospital emergency medical services team in 32% of cases. Sixty-eight percent of our patients were brought by the own facilities of their family members. One hundred and twelve patients (40%) came from Sfax city or its neighborhood; however, 60% came from other cities of south Tunisia.

**Figure 1:**
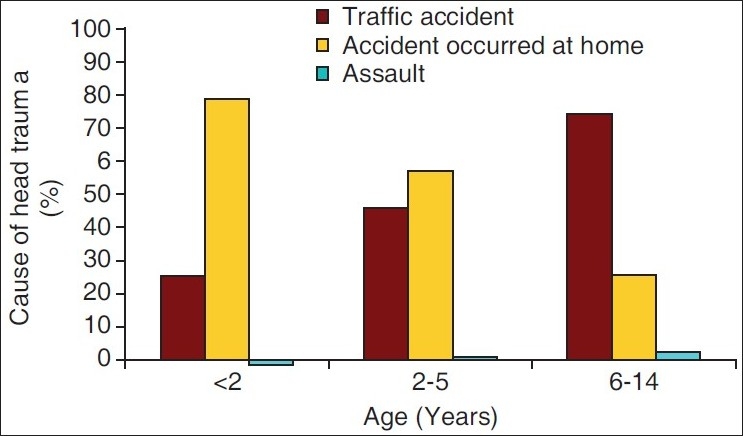
Cause of head trauma in each age groups

There were 196 male (71%) and 80 female patients (29%) with a mean age of 6.7 ± 3.8 years (range = 0.3–15). Fifteen percent of children were aged less than 2 years, 17.8% were 3–5 years old and 41% were 6–10 years old. The demographic and clinical parameters on admission are shown in Table 1. Head traumas were mainly from traffic (58.3%) and home (39.1%) accidents. However, head injuries from home accidents were commoner among younger children than older children compared to traffic accidents and *vice versa* [Figure 1]. The mean GCS score on admission was 8 ± 2 points. The head trauma was complicated by mild brain injury (GCS, 13–15) in 17.8%, moderate brain injury (GCS, 9–12) in 33.3%, and severe brain injury (GCS, 3–8) in 48.9%.

**Table 1:** Demographic and clinical parameters on admission of all study population

Parameters	Mean ± SD	Number (%)
Age (years)	6.7 ± 3.8	–
Sex M/F		196/80
PTS	4.8 ± 2.3	–
ISS	23.3 ± 5.9	–
PRISM	10.8 ± 8	
HR (beats/min)	114 ± 29	–
SBP (mmHg)	102 ± 16	–
Respiratory distress	–	10 (3.6)
Shock	–	10 (3.6)
Cardiac arrest	–	4 (1.4)
Body temperature in °C	38 ± 1.2	–
Glasgow Coma Scale Score	8 ± 2	–
Anisocoria	–	42 (15.2)
Bilateral mydriasis	–	27 (9.8)
Motor deficit	–	45 (16.3)
Convulsion	–	67 (24.3)
Pathological antecedent	–	22 (8)

HR: HEART RHYTHM; SBP: SYSTOLIC BLOOD PRESSURE; M/F: MALE/FEMALE; PTS: PEDIATRIC TRAUMA SCORE; ISS: INJURY SEVERITY SCORE; PRISM: PEDIATRIC RISK OF MORTALITY SCORE.

In our study, brain CT-scan was performed on admission for 270 patients. For the remaining six patients, MRI was performed instead because of logistic difficulties. At admission, 259 patients needed intubation, sedation, and mechanical ventilation with a mean duration of 4 ± 6 days. On admission, 69 (25%) patients needed craniotomy. The most neurosurgical intervention were evacuation of a subdural hematoma (*N* = 6), evacuation of an extradural hematoma (*N* = 30), lobectomy (*N* = 3), elevation of depressed skull fracture (*N* = 10), and decompressive craniectomy in one case.

The results of brain CT-scan are presented in Table 2. According to Marshall tomographic grading “Traumatic Coma Data Bank classification” there were 29% type I, 37.7% type II, 9.8% type III, 1.1% type IV, 17.4% type V, and 5% type VI. In this study, normal cerebral CT scans were observed in 40 patients (14.5%). During the ICU stay, 178 patients (64.5%) had complications: nosocomial infections 46 cases (16.7%), pneumonia 27 (9.7%), tract urinary infection 9 (3.2%), meningitis 6 (2.1%), septicemia 6 (2.1%), and inner ear infection or sinusitis 6 (2.1%). During the ICU stay, 101 patients (36.6%) had required fluid resuscitation. Catecholamines were used in 14 patients (5.1%) (dopamine in 12 patients (4.3%), epinephrine 5 (1.8%), and dobutamine 1 (0. 3%)).

**Table 2:** Cerebral CT-scan findings among patients

CT scan signs	Number (%)
Normal CT-scan	40 (14.5)
Meningeal hemorrhage	91 (33)
Cerebral edema	75 (27)
Cerebral contusion	98 (35.5)
Extradural hematoma	37 (13.4)
Subdural hematoma	45(16.3)
Pneumocephalus	31 (11.2)
Mass lesion	8 (2.9)
Cerebral trunk injury	6 (2.2)
Skull fracture	118 (42.8)
Depressed skull fracture	47 (17)

A total of 95 patients (34.4%) had rhabdomyolysis (CPK > 500 IU/l).[25] Hyponatremia (<130 mmol/L) was present in 76 (27.5%), hypernatremia (>145 mmol/L) in 19 (6.9%), diabetes insipidus in 5 (1.8%), stage III or IV pressure ulcer[26] in 10 (3.6%), and neurogenic pulmonary edema in 6 (2.1%).

During the ICU stay, 222 patients (80.4%) developed SSIs. Table 3 shows the frequency of each SSI. Finally, 260 (94.2%) patients developed during their ICU stay one or more organ failure.

**Table 3:** Frequency of secondary systemic insults among patients

Type of secondary systemic insults	Number (%)
Hyperthermia	126 (45.7)
Hyponatremia	76 (27.5)
Arterial hypotension	73 (26.4)
Arterial hypertension	14 (5.1)
Hyperglycemia (≥11 mmol/L)	48 (17.4)
Hypoxemia	29(10.5)
Hypercapnia (>45 mmHg)	34 (12.3)
Hypocapnia (<28 mmHg)	24 (8.7)
Hypoglycemia (<2.8 mmol/L)	6 (2.2)
Anemia (Hb < 8.5 g/dL)	32 (11.6)
Diabetes insipidus	5 (1.8)

Mean ICU stay was 5.8 ± 102 days. Forty-eight patients (17.4%) died. Regarding the time of death, the mortality percentage was 60.4% in the first 24–48 h, 23% between 3 and 7 days, and only 16.6% thereafter. Brain herniation diagnosed clinically was the main cause of mortality (62.5%), whereas the other cases of mortality were: acute respiratory distress in 8.3% and sepsis with multiorgan failure in 22%.

Among the 228 survivors, 19 patients (8.3%) had a functional motor deficit, 15 (6.6%) had subjective symptoms, and 16 (7%) had posttraumatic seizures. The Glasgow Outcome Scale performed within a mean delay at 12.7 months after hospital discharge (range = 0.5–96 months) were as follows: 48 deaths (17.4%), 2 vegetative state (0.7%), and 176 good recovery (63.8%).

Univariate analysis showed that low PTS on admission, high ISS, high PRISM, presence of shock, menigeal hemorrhage, a serum glucose > 11 mmol/L and bilateral mydriasis are associated with mortality [Table 4]. Finally, in our study, a Na+ > 145 mmol/L on ICU admission (maximal level encountered 154 mmol/L) was associated with a poorer outcome (*P* < 0.0001).

**Table 4:** Factors associated with death in univariate analysis

Parameters	Survivors (%)	Deaths (%)	*P* value
Age (years) (means ± SD)	6.6 ± 3.7	7.2 ± 4.1	0.34
PTS (means ± SD)	5 ± 2	3 ± 2	<0.0001
ISS (means ± SD)	22.8 ± 6.4	25.7 ± 1.5	0.002
PRISM (means ± SD)	8.6 ± 5.3	20.7 ± 10.6	<0.0001
Glasgow Coma Scale Score	8.8 ± 3	6.6± 2.8	<0.0001
Shock	1.3	14.6	<0.0001
Bilateral mydriasis	5.3	31.25	<0.0001
Anisocoria	12.7	27	0.1
Neuro-vegetative disorders	4.4	20.8	<0.001
Meningeal hemorrhage	28	56.25	0.001
Subdural hematoma	14.5	25	0.2
Cerebral contusion	33.8	44	0.40
Type IV lesion according to Marshall classification	0.4	27.1	<0.001
Prothrombinemia (%)	63.3 ± 16	52 ± 21	<0.0001
Hemoglobin level (g/dL)	10.6 ± 1.8	10.03 ± 1.6	0.049
HCO_3_ (mmol/L)	17.25 ± 3.5	14.8 ± 3.8	0.049
Serum glucose > 11 mmol/L	11.2	48.9	0.0003
ASAT	74.9	117	0.02
Serum Na > 145 mmol/L	2.6	27.1	<0.0001
Diabetes insipidus	0	10.4	0.0002
Secondary Systemic Insults (SSI)	78	94	0.002
Number of organ failure ≥ 3	20.6	72.9	< 0.0001

PTS: PEDIATRIC TRAUMA SCORE; ISS: INJURY SEVERITY SCORE; PRISM: PEDIATRIC RISK OF MORTALITY

The multivariate analysis showed that factors associated with a poor prognosis were PRISM > 24 (*P* = 0.001; OR = 10.98), the presence of neurovegetative disorders (*P* = 0.004; OR = 7.1), type IV lesion. According to Marshall tomographic grading (*P* = 0.02; OR = 13.2) and the presence of meningeal hemorrhage (*P* = 0.03; OR = 2.74).

A significant association was found between PRISM score and mortality rate. This model had a high discriminative power. In fact, a PRISM score > 24 was associated with death with a sensitivity of 37%, a specificity of 99.95%, and with an area under the ROC curve at 0.85 [Figure 1].

**Figure 2:**
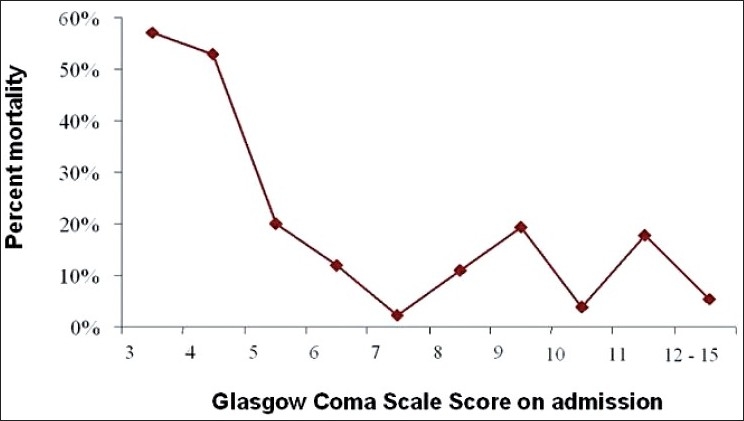
Mortality rate correlated with Glasgow Coma Scale Score

In addition, as shown in Figure 2, a low value of GCS score on admission was associated with a poor outcome. In fact, GCS score ≤ 8 was associated with death with a sensitivity of 62%, a specificity of 74%, and an area under the ROC curve at 0.70.

**Figure 3:**
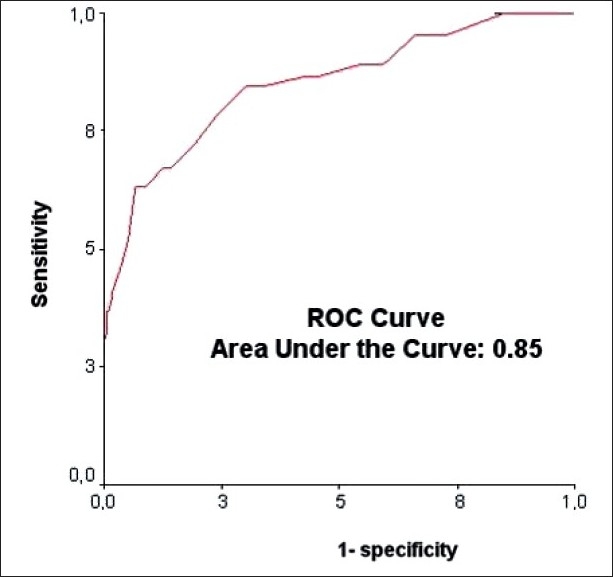
Roc curve for ability of PRISM score to predict mortality

In our study, we founded a good correlation between the PRISM score and the outcome. In fact, a PRISM score > 24 was associated with a poor outcome with a sensitivity at 37% and specificity at 99%, and an area below the ROC curve at 0.85 [Figure 3]. However, ISS and PTS are not enough discriminating with areas below the ROC curve at 0.0.62 and 0.72, respectively.

**Figure 4:**
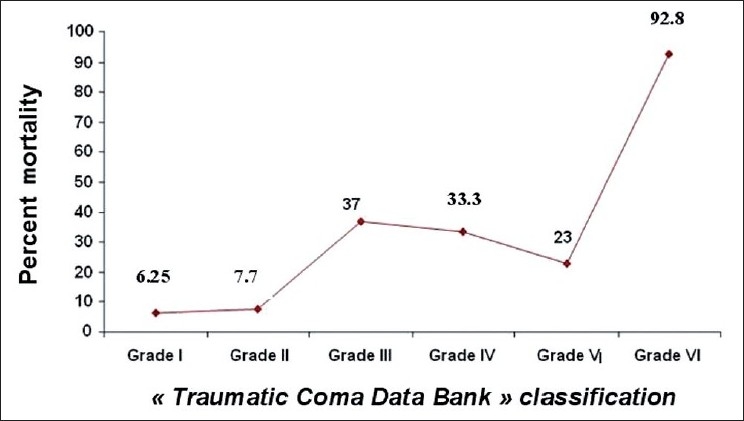
Mortality rate according to “Traumatic Coma Data Bank” classification

**Figure 5:**
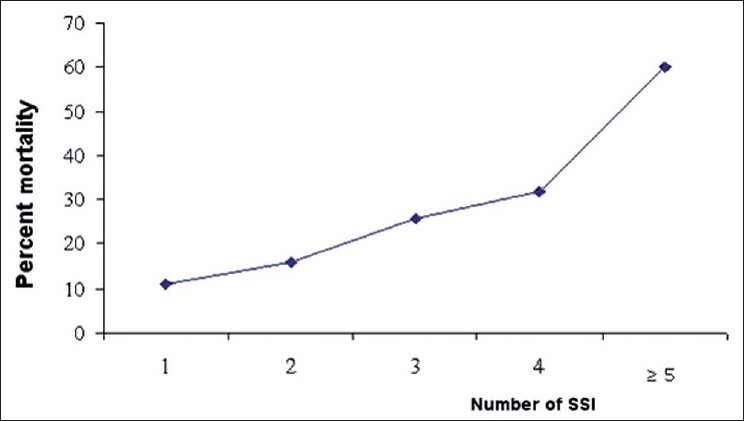
Association between mortality and the number of developed secondary systemic insults (SSI)

According to “Traumatic Coma Data Bank” classification, mortality rate was at 6.25% in type I group, 7.7% in type II, 37% in type III, 33% in type IV, 23% in type V, and 93% in type VI (*P* < 0.001) [Figure 4]. Moreover, as shown in Figure 5, the mortality rate was narrowly related with the number of developed SSIs (*P* < 0.0001). In fact, mortality rate was increased from 11.2% in patients with only one SSI to 60% in those having more than five SSI.

Finally, the development of organ failure was associated with mortality (*P* < 0.001). In fact, mortality rate was increased from 0% in patients without organ failure to 64% in those having more than four organ failures. Table 5 shows the association between of each organ failure and the mortality.

**Table 5:** Association between organ failure and prognosis

Type of organ failure		Survivors (%)	Deaths (%)	*P* value
Circulatory	Yes	39 (17.1)	33 (68.75)	<0.0001
	No	188 (82.9)	15 (31.25)	
Respiratory	Yes	206 (90.3)	48 (100)	0.02
	No	22 (9.7)	0 (0)	
Neurological	Yes	120 (54)	44 (91.7)	<0.0001
	No	105 (46)	4 (8.3)	
Haematological	Yes	22 (1)	13 (27.1)	0.004
	No	205 (89.9)	35 (72.9)	
Renal	Yes	0 (0)	3 (6.25)	0.001
	No	227 (99.5)	45 (93.75)	
Liver	Yes	24 (10.5)	9 (18.75)	0.2
	No	203 (89)	39 (81.25)	

## DISCUSSION

Head injuries occur commonly in childhood and adolescence. Most head injuries are mild and not associated with brain injury or long-term complications. Very rarely, children with more significant injuries may develop serious complications (e.g., brain injury or bleeding around the brain).[27,28] Studies have shown that trauma is the leading cause of death among children aged 5–15 years in the developed and developing countries with traffic accidents being the predominant cause of fatal injuries in children.[27,28] In this study, traffic accident was the commonest cause of head injuries among the study cohort. Despite the unavailability of specific monitoring tools (jugular venous saturation, intracranial pressure monitoring, and transcranial Doppler sonography) in our ICU, the mortality rate was of 17.4%, which is somewhat similar to those reported in other studies[4,29,30] and in particular in developed countries.[29–31] Several investigators have stated that age is a good indicator of mortality in traumatic brain injury.[7] Mortality is higher at extreme ages of life.[7,31] The influence of age on outcome of children with severe head injury is controversial.[31–34] As in other studies,[29,30] this factor was not significant in our series (*P* = 0.34). However, we cannot conclude that age has no effect on outcome. Our findings can be explained by the fact that all children were not managed similarly. In fact, our study is retrospective with a period of 8 years. Before January 2000, barbiturate therapy (thiopental 50 mg/kg/day) were used for all patients suffering severe head injury and requiring mechanical ventilation; nevertheless, this therapy was abandoned since that period.

The GCS Score is used widely as a guide to the severity of brain injury.[8] Several studies have shown that there is a good correlation between GCS Score and neurological outcome.[7,29,31] In our series, neurological state was assessed using the GCS Score at the site of accident and again on hospital arrival before the use of sedative but after resuscitation: the preintubation GCS (used in our analysis) and we found that GCS Score was associated with mortality only in univariate analysis (*P* < 0.001).

The standard of care for any patient with TBI includes serial neurologic examinations. These examinations include a pupillary assessment and are often performed by trauma nurses at the scene in the emergency department and in the acute and critical care units.[35,36] A key component of any neurologic assessment is the pupillary examination, and pupil reflexes recovery is a useful predictor of recovery after brain trauma. When an abnormality is detected, the trauma nurse should first identify whether the abnormality was present on the previous pupillary examination. If an abnormal pupil is present on the initial pupillary examination, it should be clearly documented, and a physician should be immediately notified. Immediate notification of a physician should occur with changes in pupillary response. Comparing the current examination with the previous to provide time-oriented data for the physician is wise but should never delay immediate physician notification. Fearnside *et al*.[37] showed a significant difference in mortality according to whether both pupils reacted or not (*P* < 0.05). In fact pupil reactivity is related to cerebral blood flow.[36] In our study, bilateral mydriasis was clearly associated (*P* = 0.01) with mortality in the univariate analysis. In the ICU, these indicators contribute to an objective evaluation of outcome. The ISS is a commonly used scoring system in traumatology.[38] Some studies have not found ISS to be a good outcome predictor, even in cases with serious injuries.[39] Others have found it to be a good predictor of poor prognosis.[35,40] We demonstrated a significant relationship between the ISS and the prognosis in univariate analysis.

The PRISM score has been widely used as a severity score in critically ill children in various clinical situations.[11] However, only few studies using the PRISM score in pediatric trauma patients are available.[38] These studies showed that PRISM was an accurate tool for predicting outcome.[34,38,41] In our study, we showed with multivariate analysis that PRISM score was a reliable tool for predicting death, as demonstrated by the high value (0.85) of the area under ROC curve. Our findings are in agreement with previous studies.[11,38,42]

The CT-scan is also assumed to reflect the seriousness of head injuries and predict clinical course. The influence of the type of cerebral lesions on cranial pressure and mortality has been evaluated variously in the literature.[43,44] However, some lesions appear to carry a poor prognosis: in our study, the presence of meningeal hemorrhage was associated with mortality only in univariate analysis. Eisenberg *et al*.[14] found a good relationship between such lesion, increased intracranial pressure and death in a study of 753 cases with severe head injury, and the presence of these lesions may appear to double mortality in two otherwise comparable groups. An explanation could be ischemia which may cause reduced cerebral blood flow in the acute stages, and then after a slight increase to levels near the lower limit of normal range in the subacute stages.[45] In our study, meningeal hemorrhage was associated with high mortality. In addition, according to the “Traumatic Coma Data Bank” classification, the mortality rate was significantly higher in patients with type VI lesions. This “Traumatic Coma Data Bank” classification differentiates between patients with and without mass lesions. Since its introduction, this CT classification has become widely accepted for descriptive purposes, and is also increasingly being used as major predictor of outcome in traumatic brain injury in adult’s patients.[47,48]

The negative influence of the secondary systemic insults to the brain (SSI) is well documented. In our study like other studies,[49–50] the development of SSI was associated with a poor prognosis in univariate analysis. In addition, the mortality rate is greatly associated with the number of SSIs.

A significant correlation between glucose levels, severity of head trauma, pupillary reaction, and maximum intracranial pressure during the first 24 h after trauma was well documented.[49–50] Our study shows that high level of blood glucose on admission was associated with a poor outcome. This finding was previously reported in other studies conducted in our ICU.[49–50]

Despite the initial severity of our patients’ conditions (94% of them needed intubation and mechanical ventilation) and the lack of specific monitoring tools (intracranial pressure monitoring, transcranial Doppler sonography, and jugular venous saturation), the mortality rate (17.4%) in our study seems to be within the literature standards. Our series may somewhat suggest that the overall rate of mortality may not be different with or without invasive monitoring. Despite the retrospective nature of this study, we were able to define some simple variables that are predictive of a poor short-term prognosis and that are merely based on easily measurable clinical, CT scan, biochemical and laboratory parameters that may be used either at the scene of the accident (clinical) or in the emergency department of any hospital with available facilities. According to the National Guard Statistical Data, seatbelt is used only in 22% of car conductor and only 60% of motorbike drivers wear helmet in our population.[5] The majority of motorcycle-related deaths and hospital admissions are caused by head injuries. The use of motorcycle helmets reduces the risk of head, and brain injuries for bicyclists of all ages. Thus, all motorbike riders and passengers of all ages should wear helmets every time they ride motorcycles. The use of booster seats as well as the correct installation of children is recommended. Furthermore, children should be taught how to safely cross the road especially to stop at the curb and watch either sides of the road before crossing. Young children should never cross the street alone. Moreover, improved pre-hospital care, a readily available multidisciplinary emergency teams, the establishment of regional trauma centers, and efforts to prevent motor vehicle crash should improve the poor prognosis of severe head injury in children. Head injuries can often be prevented.

Finally, we must mentioned that our study have some limitations. First, all retrospective studies as our study suffer from incomplete or inconsistent information. Second, the retrospective nature of the case series makes it difficult to envision producing extensive analysis. Furthermore, in our ICU the diagnosis of raised ICP was performed on clinical (pupil response, motor deficit, and convulsion) and brain CT-scan results; however, we think that the inability to measure ICP adversely effects our ability to assess ICP. Our findings concerning functional outcome according to the Glosgow Outcome Scale suggest that even an initial “good recovery” can be observed, it can be followed by poor long-term functional outcome. In fact, as the child grows up; sequelae squeals of traumatic brain damages become more obvious with severe handicap.

## CONCLUSION

Motor vehicle crashes represent the main etiology of severe head injury among children. Short-term prognosis of head injury is poor and prognosis in a head-injured child who is still alive cannot be determined at the roadside.
